# Choosing the Narrative: the Shadow Banking Crisis in Light of Covid

**DOI:** 10.1007/s11079-020-09614-2

**Published:** 2021-02-11

**Authors:** Marcus Miller

**Affiliations:** Department of Economics, University of Warwick, Coventry, CV47AL UK

**Keywords:** Shadow banking, Rating agencies, Equity constraints, Technology and news shocks, Bank runs, Epidemic and social contagion, G01, G24, G41, Y8, Z13

## Abstract

Could experiencing a health pandemic aid in understanding the nature of financial crisis? It might, for example, help to discriminate between different narratives that claim to do so. In this spirit, two influential accounts of the near-collapse of shadow banking in the US financial crisis of 2008 are analysed: one developed by Mark Gertler and Nobuhiro Kiyotaki and the other presented by the Financial Crisis Inquiry Commission of the US Congress. Using a common two-sector framework, key features of these contrasting accounts of the market for banking services are presented, along with their corresponding diagnoses of what precipitated financial crisis. To see what the experience of Covid might imply about their relative credibility, four aspects of the current pandemic are considered: how it began from a small biological shock; how it gets spread by contagion; the significance of externalities; and how it may end with a vaccine. But the reader is left to form his or her own judgement.

## Introduction

In “The Jungle Tale and the Market Tale”, one of his *Economic Fables,* Rubinstein (2012) offers two accounts of how an economy might allocate resources – by the ‘iron hand’ of relative strength; or by the ‘invisible hand’ of competitive equilibrium. In both cases, he promises, ‘I will follow economic tradition and demonstrate the ideas via models – tales or fables^1^ … But I will not use formal language, which would make the models easier to understand for the few who are familiar with this language, but would pose an impenetrable barrier for all the rest.’

It is in this spirit that two influential accounts of near-collapse of shadow banks in US financial crisis of 2008 are examined here. This is not intended, however, as an intellectual exercise of comparing market and non-market mechanisms for allocating resources. Rather it is to see what we might learn from the health crisis - by comparing two influential but sharply contrasting accounts of the market for banking services and of what went wrong in the financial crisis; then seeing what the current experience of the Covid pandemic might imply about their relative credibility.

Four aspects of the current pandemic are considered: how it began from a small biological shock; how it gets spread by contagion; the significance of externalities; and how it may end with a vaccine. But the reader is left to form his or her own judgement.

### Contrasting Narratives

One of the narratives to be discussed has been developed by distinguished academics, Mark Gertler of Columbia University and Nobuhiro Kiyotaki of Princeton University, in a series of technical papers appearing in leading economic journals and handbooks since 2015. This, the Scholars’ Tale, holds that US shadow banks^2^ provided highly efficient financial intermediation – with lower spreads between expected asset returns and borrowing costs than commercial banks, for example. Following hard on the heels of an unanticipated negative productivity shock, however, the financial sector suffered a systemic ‘bank run’, attributable to a ‘sunspot’ – a metaphor for some random, pay-off irrelevant, extrinsic trigger. In short, shadow banks are portrayed as efficient but fragile institutions exposed to extraneous random shocks for which they bear no responsibility.

A starkly different picture is painted in the majority report of the Financial Crisis Inquiry Commission (FCIC) set up by US Congress to investigate the origins and course of the crisis. It stresses the significance of information asymmetries, and the disingenuous role played by the Credit Rating Agencies (CRAs) in particular. Early in the *Final Report*, FCIC ( 2011, p.xxv) one reads:

The three credit rating agencies were the key enablers of the financial meltdown. The mortgage-related securities at the heart of the crisis could not have been marketed and sold without their seal of approval. … Their ratings helped the market soar and their downgrades through 2007 and 2008 wreaked havoc across markets and firms. From 2000 to 2007 Moody’s rated nearly 45,000 mortgage related securities as triple-A. This compares with six private sector companies in the United States that carried this coveted rating in early 2010.

Specifically, the FCIC alleges that competition for business between different rating agencies enabled investment banks to secure unrealistically optimistic ratings for assets that they held and sold; and that this overvaluation played a key role in the ensuing crisis.

This, the Congressional Tale, later received academic support from George Akerlof and Robert Shiller as a case study showing how those with better information can profit at the expense of the less well-informed.^3^ In the push to provide credit to subprime borrowers, they argue, investment banks, protected by the shield of limited liability, were tempted to assemble highly-rated but very risky securitised assets for sale to other investors and to hold in their own portfolios. Crisis arrived when such practices came to light. Unlike the ‘sunspot’ model of banking crisis endorsed by Gertler, Kiyotaki and co-authors, the account of Akerlof and Shiller is concerned with the formation of beliefs in a setting of imperfect information.

### What Is to Follow

Could these narratives of crisis not first be compared one against the other – in a type of intellectual beauty contest where contestants seek to be most persuasive? To this end, the stage is set in the next section by outlining a basic two-sector framework, where shadow banks using borrowed funds compete with non-bank ‘direct’ lenders, leading to a market equilibrium with substantial intermediation. In section three comes the beauty contest, where each narrative offers its own distinctive twist to the framework, and shows how this can lead to banking collapse. In section four, the light that experience of the current medical epidemic may throw on these competing narratives of financial crisis is considered. Section five concludes.

## A Basic Framework to Set the Scene

**Fig. 1:**
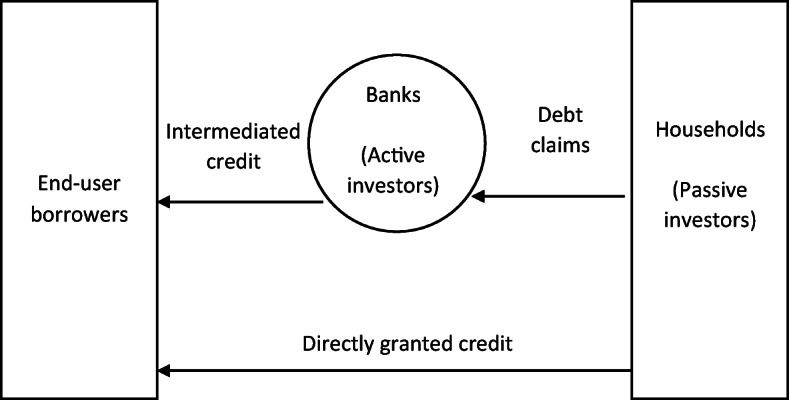
Intermediated and directly granted credit

The basic framework to be used involves competition between ‘direct’ lending and ‘intermediated’ lending by shadow banks to satisfy the needs of the ultimate borrowers. This is shown schematically in Fig. 1 from Shin (Shin 2010, p.30), where the securities issued by the ultimate borrowers (on the left) are taken up by risk-neutral shadow banks, relying heavily on borrowed funds, and other non-bank holders (on the right) who use their own resources and are risk averse.

**Fig. 2:**
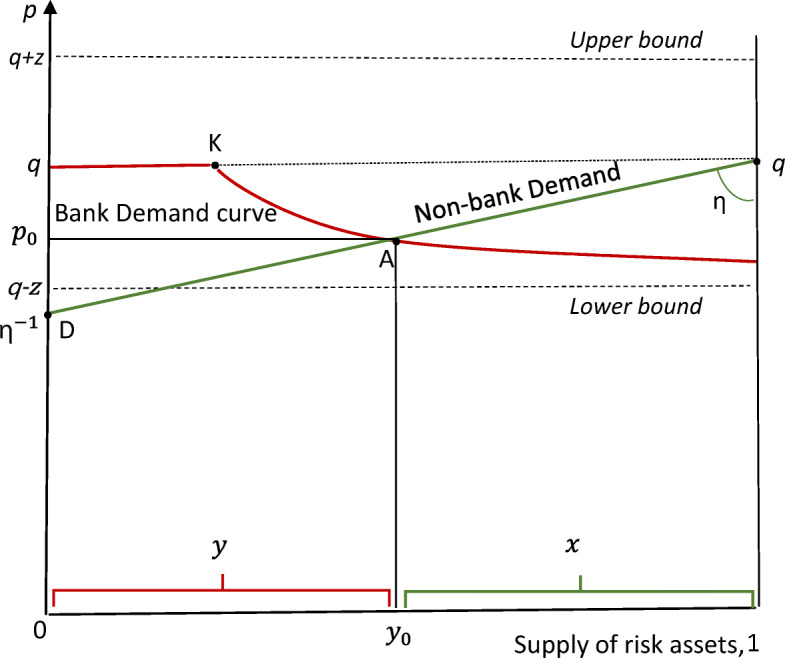
The price of risky assets determined by market-clearing

Taking the amount of risky assets issued by end-user borrowers to be fixed, the market outcome proposed by Shin himself is illustrated^4^ in Fig. 2 below. Payoffs for the risky asset are assumed to have a uniform distribution between the upper and lower bounds shown, with mean return *q* and downside risk *z*. The combined demand by both sectors determines the price of risky assets, p, measured on the vertical axis.

The demand curve for banks, assumed to maximise the expected value of their holdings subject to an equity constraint to prevent risk-shifting, is measured from the left-hand axis. Given the specific Value at Risk (VaR) rule that ‘equity, *e*, must cover all downside risk to which the portfolio is exposed’, bank demand for *p* ≤ *q* is in fact shown by the rectangular hyperbola passing through K (asymptotically approaching the vertical axis and the lower bound of risky payoffs as *y* = *e*/(*p* − (*q* − *z*) . There is, of course, no demand at prices standing above the mean expected payoff.

Graphically, the kink at (*q*, *e*/z), labelled K, indicates the volume of assets with downside risk *z* covered by equity when price is at the mean; and the downward slope thereafter indicates, not risk aversion, but the effect of adhering to the VaR rule.^5^ The demand from the other, risk-averse sector, measured from the right-hand axis, increases in proportion as the price falls below *q* (i.e. *x* = *η*(*q* − *p*) with slope,−1/*η*, flatter the higher the tolerance for downside risk^6^). Market-clearing equilibrium is at A, where the schedules intersect at price *p*_0_.

Since the equilibrium price lies ‘within the band’ between *q* and *q* − *z*, banks will expect to make a positive Return on Equity (ROE), especially if no or low interest is paid to creditors who bear no risk^7^ (given that equity covers the total risk exposure on the balance sheet). That shadow banks can hold risky assets while issuing money-like liabilities is the so-called alchemy of finance – sustained by shadow bank shareholders shouldering substantial risk.

The framework just described effectively assumes Common Knowledge: so, as portfolio managers in both sectors know the true values of parameters such as *q* and *z* (and know that others do), there is strictly speaking no role for credit rating agencies. In the concluding chapter of *Risk and Liquidity,* however, Shin indicates that, in practice, things are not so straightforward. For credit ratings are widely used; yet, one is warned, ‘heavy reliance on credit rating agencies … is misguided. They are unregulated and the quality of their risk estimates is largely unobservable’ Shin (2010, p. 171). Taking our cue from this remark, we turn next to the perspective of the FCIC with its focus on issues of asymmetric information.

## A Beauty Contest: The Narratives Compared

### The Congressional Tale: Of Fooling, Revelation and Panic

As a preliminary, we note that, for Bolton and Dewatripont (Bolton and Dewatripont 2005, p. 175), a ‘central result in the literature on disclosure of information is that when certification is costless, there is full disclosure of information in equilibrium under very general conditions.’ So it might be thought that the existence of well-established credit rating agencies – and the active role that they play in US securities markets^8^ - could have ensured that issues of information asymmetries were largely finessed - with conservative banks borrowing at low rates of interest compared with risky banks paying much higher rates, for example. But if, as alleged by the FCIC Report, ratings are the outcome of competition for business between unregulated oligopolists - no longer run as partnerships but as profit maximising corporations^9^ - then full disclosure of information is unlikely. Flattering assessments of product quality, which promote the profits of clients who pay for the ratings, are a tempting way of keeping them on the books; and, the Commission concluded, “the rating agencies placed market share and profit considerations above the quality and integrity of their ratings.” FCIC ( 2011, p. 212).

#### A ‘Fooling’ Equilibrium that Unravels

By its own account, the Congressional Commission ‘interviewed more than 700 witnesses, held 19 days of public hearings, and examined millions of pages of documents’; and its majority Report runs to some 400 pages. Though no formal model is offered, the tale that is told may be illustrated by reinterpreting the basic framework spelled out above.

What the tale implies, however, is that an equilibrium in the market for risky securities seemingly based on Common Knowledge should be treated with caution. If behaviour was based on false certification, it will instead lead to a type of ‘fooling equilibrium’^10^ where creditors who lend to shadow banks holding highly-rated subprime securities underestimate the true risks being taken with their funds, and fail to realise their own exposure until too late.

Why banks should wish to get the risky assets they hold - and assemble for sale - over-rated is not far to seek: it is a way of exploiting the shield of limited liability. For over-rating is a way of working around the VaR rules, shielding shareholders by exposing creditors to significant downside risk. With more expected profits to be made on the upside, but extra losses transferred to creditors on the downside, hidden risk raises the expected ROE for the banks – assuming creditors also believe the rosy ratings.^11^

**Fig. 3:**
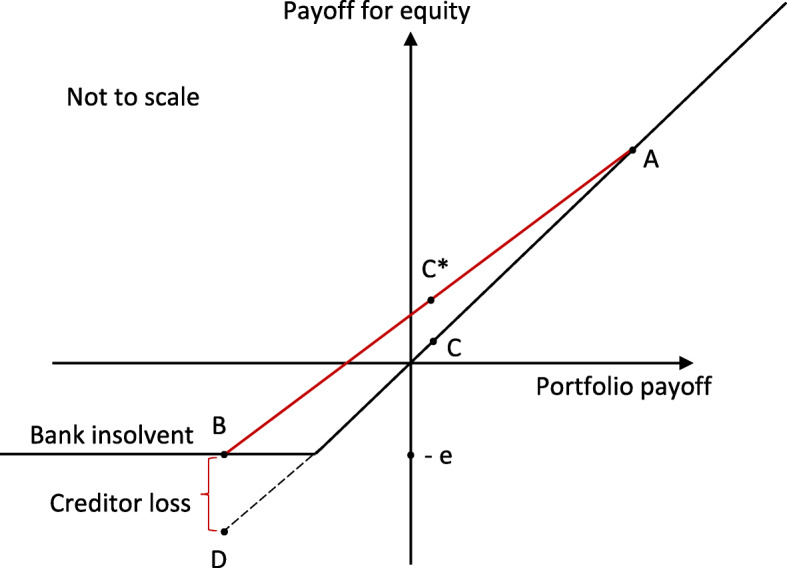
With limited liability: ‘gains we keep, losses we share’

It is the contention of Goodhart and Lastra (2020), indeed, that “the limited liability of equity holders is by far the biggest source of moral hazard and risk shifting in a capitalist economy”; and the diagram from their paper, see Fig. 3, conveys this critique most cogently.^12^ Even though there is a safe investment that gives a certain return of C to equity holders, they can expect to do better if the bank invests in risky securities - as, for example, one with the same mean but two equi-probable payoffs, A which is highly profitable and D where losses will drive the bank into insolvency. With liability limited to *e*, equity invested, losses on the downside are shared with the creditors, as shown; so the expected payoff to equity rises from *C* to *C*^∗^ - but only so long as the creditors are ‘fooled’.

If ‘at the length truth will out’, then such fooling equilibria will be liable for correction. What to expect when the true quality of the risk assets is revealed – i.e. after what Bernanke (2018) describes as ‘bad news’ arrives? The basic framework – suitably reinterpreted - can provide a simple account of the Congressional Tale.

**Fig. 4:**
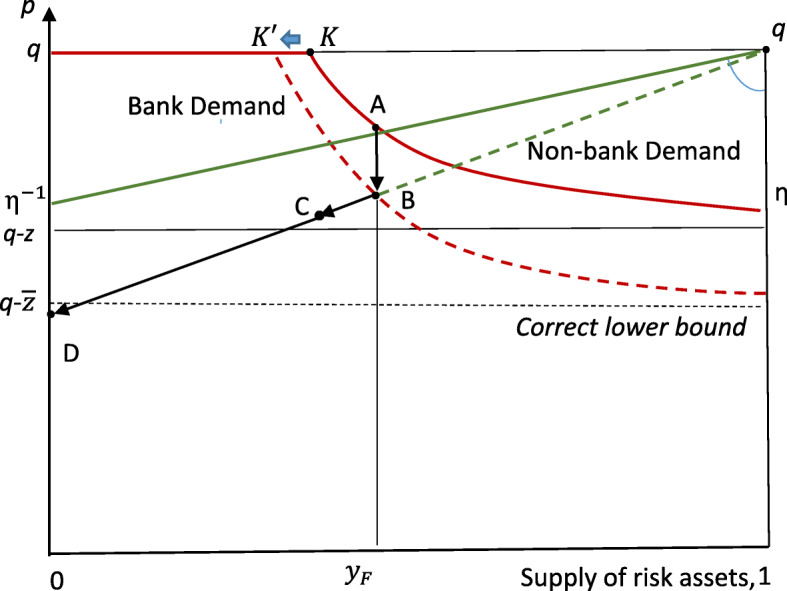
Effect of unanticipated Bad News: on impact and subsequently

^13^

If the initial equilibrium was based on an under-estimate of the actual downside risk, then the immediate effects of realising the truth are shown in Fig. 4. As before, equilibrium is determined by market clearing at A, but *this is now interpreted as a ‘fooling’ equilibrium, y*_*F*_*, based on an underestimate of the riskiness of the assets involved.* The increase of perceived risk when the truth is revealed will make these assets less attractive to both sectors. For passive investors the rise in downside risk (from *z* to $\overline{z}>z$) makes their demand schedule steeper, as indicated by the anti-clockwise movement in the figure. The demand curve for shadow banks, subject to a binding VaR constraint, shifts to the left (from K at *e*/*z* to K′ at $e/\overline{z}$ at the top of the figure) as the unit risk increases to its true value; and becomes steeper as the lower asymptote moves down to $q-\overline{z}$, the correct lower bound. Hence equilibrium will, on impact, move from A to B as shown, with a fall in the price but not much trading of assets.

This reduction of demand is not the end of the story, however. With assets ‘marked-to-market’ at lower prices, the fall in shadow bank equity permits less asset-holding as the VaR constraint binds tighter. This endogenous adjustment of bank equity will amplify the effect of Bad News, with investment banks losing market share as equilibrium shifts along the demand curve for passive investors to a point like C.

Will these mark-to-market effects lead to insolvency of shadow banks? Not if, as shown, the falling price still lies within the range for which equity provision was made (i.e. C lies above *p = q-z*). But this is without taking into account the reaction of creditors.

#### Creditor Panic

In his account of the crisis, Ben Bernanke (2018) describes how creditors respond to bad news:

Before the crisis, investors (mostly institutional) were happy to provide wholesale funding, even though it was not government insured, because such assets were liquid and perceived to be quite safe. Banks and other intermediaries liked the low cost of wholesale funding and the fact that it appealed to a wide class of investors. Panics emerge when bad news leads investors to believe that the “safe” short-term assets they have been holding may not, in fact, be entirely safe. If the news is bad enough, investors will pull back from funding banks and other intermediaries, refusing to roll over their short-term funds as they mature. As intermediaries lose funding, they may be forced to sell existing loans and to stop making new ones.

How to factor in such behaviour? For commercial banks, creditor panic takes the form of a bank run with customers withdrawing deposits; but for non-deposit-taking institutions like shadow banks, the funding squeeze shows up instead as a sharp rise in the ‘haircut’ imposed on collateral for repo borrowing.^14^ As Shin notes, ‘fluctuations in the haircut largely determine the degree of funding available to a leveraged institution … [and] times of financial stress are associated with sharply higher haircuts’ (Shin 2010, p. 144, 145).

Just how much higher Shin illustrates by the rise in haircuts from April 2007 before the crisis to August 2008 in its midst, so ‘a borrower holding AAA-rated residential mortgage backed securities would have seen *a tenfold increase in haircuts* [from 2% to 20%], meaning its *leverage must fall from 50 to just 5’* (emphasis added). Here is market evidence of a systemic bank run of such dramatic proportions as to precipitate prompt insolvency of the shadow banks,^15^ absent official support.

The crisis account of the FCIC can, it seems, be encompassed by appropriate reinterpretation of the basic framework, as in Fig. 4. When A, interpreted as a ‘fooling equilibrium’ (where creditors are lulled into a false sense of security by inflated ratings) comes to an end, with asset prices falling as indicated by B, then shadow bank portfolios contract as their equity takes a hit, see point C. This generates visceral panic as creditors wake up to the risks they are exposed to - by banks whose liability is strictly limited to the loss of their own (now reduced) equity – leading to collapse of shadow banking (as prices fall beyond what their equity can cover), as at D in the figure.^16^

To put it bluntly, the Congressional narrative involves what Akerlof and Shiller (2015) characterise as ‘the economics of manipulation and deception’; with crisis as the denouement.

### The Scholars’ Tale: Of Efficiency, Productivity Shocks and Sunspots

Before getting into detail, here is how shadow banking is viewed in the tale told by these academics:

In “normal” times, the growth of the wholesale banking sector improves both efficiency and stability. Improved efficiency stems from the comparative advantage that wholesale banks having in managing certain types of loans. Improved stability arises because retail banks act as a buffer to absorb loans that wholesale banks sell off, in effect improving the liquidity of secondary loan markets.

On the other hand, the growth of wholesale banking system makes the economy more vulnerable to a crisis. As occurred in practice, the high leverage of wholesale banks makes this sector susceptible to runs that can have highly disruptive effects on the economy. A contractionary disturbance that might otherwise lead to a moderate recession, can induce a run on the wholesale banking sector with devastating effects on the economy, as experienced during the Great Recession. Gertler et al. (2016)

The FCIC did not provide their own technical analysis; but the opposite is true in this case. Three key papers relevant here run to many pages, and the number of equations increases as the model is extended. (From 27 in Gertler and Kiyotaki (2015) with a single bank sector, reaching 39 when wholesale and retail banks are treated separately, Gertler et al. (2016), and more with the addition of a macro model in Gertler et al. (2020)). Although the models with wholesale and retail banks treated separately are more revealing, it is clearly impossible to do them justice in a short piece. The key features of banking and its crises are common to all three papers, however, so we focus on the earliest paper where, in any case, ‘the banking sector … corresponds best to the shadow banking system which was at the epicentre of the financial instability during the Great Recession’, Gertler and Kiyotaki (2015, p. 2016).

Can the results the authors obtain be expressed in terms comparable with the two-sector framework with which we started? In broad-brush terms they can: for here too bank intermediation has to compete with ‘direct’ lending by households; and banks are, likewise, so efficient that they could satisfy the needs of all borrowers were it not for the limitation of an equity constraint. And the workings of the model can be illustrated from the numerical results the authors provide.

One key difference to note, however, is that here the equity constraint is not designed to check portfolio risk-taking. The anticipated return on the liabilities of end-use borrowers is perceived as non–stochastic; so, except for unanticipated shocks, *lending involves no risk*! The competitive edge of bank intermediation lies not in its capacity to take on risk but in the assistance it can provide to enable borrowers achieve potential gains in productivity. Banks can do this with unflagging efficiency, but for non-banks (households) there are ‘management costs’ reflecting their lack of expertise in screening and monitoring investment projects; and these costs increase more than in proportion with the amount of assets being handled in the non-bank sector.

**Fig. 5:**
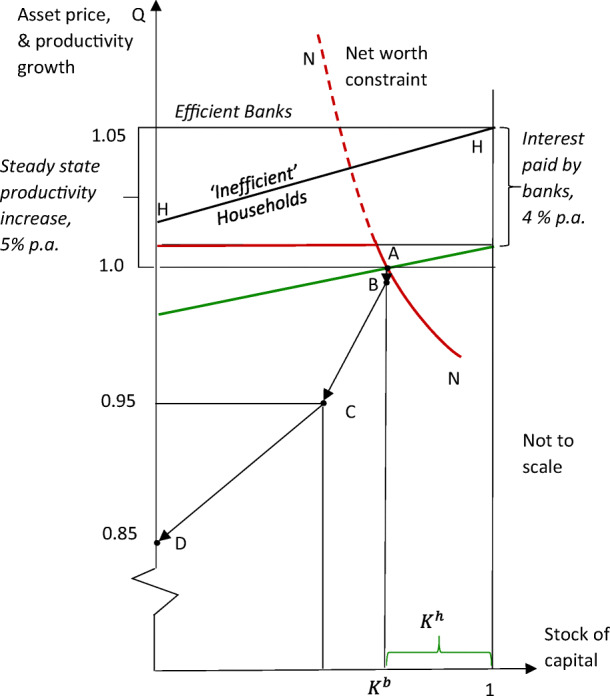
Key elements of Gertler and Kiyotaki (2015): a productivity shock followed by a bank run

This is illustrated in Fig. 5 below, based on the reported calibrations, with expected productivity growth of assets (‘capital’) of all borrowers measured in percent per annum on the vertical axis. The productivity payoff for those holding bank-intermediated assets is measured from the left hand axis (as in Figs. 2 and 4), and for household-managed assets measured from the right. With bank intermediation, *all* borrowers could expect to achieve the national rate of productivity increase – whose steady state value is calibrated at 5% p.a. in the simulations. While a few households can match this on their own, efficiency diminishes as non-bank handling of assets expands, as indicated by the schedule HH, with calibrations suggesting the least efficient household will achieve less than half potential productivity growth that banks can deliver.

In this model of riskless intertemporal optimisation - unlike the basic model of section 2 where perceived asset risk gives rise to liquidity preference - shadow banks pay generous interest rates on their liabilities^17^; and households discount asset yields by the rate of time preference. So, as shown in Fig. 5, the steady-state asset demands for both sectors are shifted below expected payoffs by the rate of interest paid on ‘deposits’, which matches the rate of time preference. As bank demand is also subject to a net worth constraint,^18^ the pre-crisis equilibrium involves direct financing, as shown at A in the figure, where the asset price is unity and banks take about two thirds of the market.

If there are no foreseeable investment risks to be considered, why the equity constraint? The rationale provided is the danger of bankers misbehaving – not by getting the assets they manage falsely rated, but by walking away with them! Thus, in this narrative, the equity share of funding required by the market is not to prevent bankers exploiting the convexity of payoffs with limited liability, but to check the simple venality of those managing other people’s money, Gertler and Kiyotaki (2015, p. 2019). As dishonest bankers will soon get found out, however, (and depositors ‘force the intermediary into bankruptcy at the beginning of the next period’), the banker will lose the equity (the franchise value) that will fund his or her retirement. Willingness to play straight can credibly be signalled, therefore, by providing an appropriate fraction of funding from own resources – i.e. by the banker putting enough ‘skin in the game’. How much equity is required for this purpose is revealed by the leverage ratios actually observed, judged to have done the job.

#### An Unanticipated Productivity Shock

Evidently, the shock that leads to crisis cannot be a widening of the perceived risk of returns on lending – no risk is perceived ex ante. Here the shock is an unanticipated, nationwide fall in the rate of productivity growth, *Z*_*t*_, the payoff expected from bank lending. The fall is calibrated as ‘a negative 5% shock to productivity *Z*_*t*_’ – i.e. an aggregate ‘technology’ shock that reduces growth achievable at an annual rate by a modest quarter of 1 % (as 0.05 × 0.05 = 0.0025). It is assumed, moreover, that this will be reversed over time as the technology variable *Z*_*t*_ follows a deterministic, autoregressive recovery process after the shock.

In circumstances described, the effects that follow are the results of a small, zero-probability technology shock. What are these effects? As calibrated, the productivity shock - powerfully amplified due to bank leverage - has a substantial impact on the aggregate bank balance sheet; calibration shows the asset price falling by 5%, as indicated by the point C; and - with bank equity apparently halved - the banks’ share of the market, *K*^*b*^, falls by about a quarter.^19^

#### A ‘Bank Run’

Even though the productivity shock is expected die away over time (with no repeat anticipated), this is not the end of the story. For, given the effects shown at point C, including the large drop in bank equity, a bank run becomes feasible - and in the calibration that is what occurs. So, as in Cole and Kehoe’s (2000) account of self-fulfilling sovereign debt crises, with a sunspot there is a systemic bank run, where banks are wiped out and their assets transferred to the other sector, shown as the move from C to D in the figure.

The part played by the productivity shock is to fulfil the technical condition for a run, namely that bank assets - evaluated after wholesale transfer to household management – are worth less than outstanding credit. So it is not, as Bernanke put it, that ‘bad news leads investors to believe that the “safe” short-term assets they have been holding may not, in fact, be entirely safe’: for in this narrative all assets are viewed as riskless! It is, rather, that the shock increases the potential consequences of creditor coordination failure.

#### Summary

The narrative here is of financial intermediation leading to greater efficiency in managing capital assets, but a dangerous lack of resilience. The only factor that prevents banks from taking all business from households, indeed, is the ‘friction’ of an equity component in their finance needed to prevent corporate theft. Bankers do not misbehave; but they are nevertheless exposed to the vagaries of technology elsewhere in the economy.

In Gertler et al. (2016), where shadow and commercial banks are treated separately (and referred to as wholesale and retail respectively), the authors conclude:

Another important area for further investigation involves the modeling of the growth of wholesale banking. Our approach was to treat this growth as the product of innovation as captured by a reduction in the agency friction in inter-bank lending markets. Among the factors we had in mind that motivate this reduction is technological improvements that permit less costly monitoring, such as the development of asset-backed securities and repo lending.

Thus, in this narrative, the expansion of shadow banking before the crisis, far from being a move to a ‘fooling’ equilibrium, is seen as the fruit of efficiency-enhancing technological and product innovation in banking – with the benefits snatched away in a crisis brought on by extraneous shocks.

## The Covid Connexion?

What light might Covid throw on the relative plausibility of these two narratives? One a highly-technical professional assessment that shadow banks were delivering efficient intermediation services, until borrowers are hit by a zero-probability productivity shock and creditors proceed to panic. The other from a broad-based Congressional Committee sceptical of self-regulation and open to the idea of market manipulation – particularly by shadow banks and rating agencies which had recently moved to limit their liability (with Goldman Sachs going public in 1999, for example, and Moody’s in 2000).

Four aspects of the current pandemic seem relevant here: how it began from a small biological shock; how it gets spread by contagion; the significance of externalities; and how it may end with a vaccine.

### Small, Unanticipated Shocks with Large Effects

Some say that a civet cat in a wet market in Wuhan may have provided the Covid virus with a link from bats to humans – leading to devastating effects on health and the economy across the world. Who knows? But if such a small, random event could conceivably have such large effects, does this not lend substantial support to a narrative that attributes financial crisis to a small, unanticipated productivity shock?

A lot depends on what is covered by the term productivity shock. For shadow banks, it actually refers to *unexpectedly bad portfolio payoffs*. But, well before the financial crisis, Rajan (2005) had warned that financial developments might be making the world riskier - as investors took on ‘tail risk’^20^ in particular. If so, such portfolio shocks could well be endogenous - the result, perhaps, of portfolio managers without special skills mimicking the returns of ‘alpha’ traders and paying for this by taking on low probability risk of catastrophic loss.^21^

On one interpretation, therefore, the productivity shock could refer to a nationwide hiccup in technical progress; on another, it could match the Congressional narrative of hidden investment risk, with banks effectively following a strategy to ‘get these securities rated high and quit when you’re hit’.^22^

### The Process of Contagion

If the convenient but heroic assumption of Common Knowledge is dropped (as in the FCIC narrative), where do market participants get their information from? The Credit Rating Agencies have been discussed as one - none too reliable - source. Could it be that ideas are also disseminated in a process of ‘social contagion’ rather like a virus, as suggested by Robert Shiller when writing about the subprime crisis in 2008. What relevance might this have to the crisis in shadow banking?

According to Shiller (2008, p. 29) “The housing bubble was a major cause, if not *the* cause, of the subprime crisis… The perception that real estate prices could only go up, year after year, established an atmosphere that invited lenders and financial institutions to loosen their standards and risk default.”

**Fig. 6:**
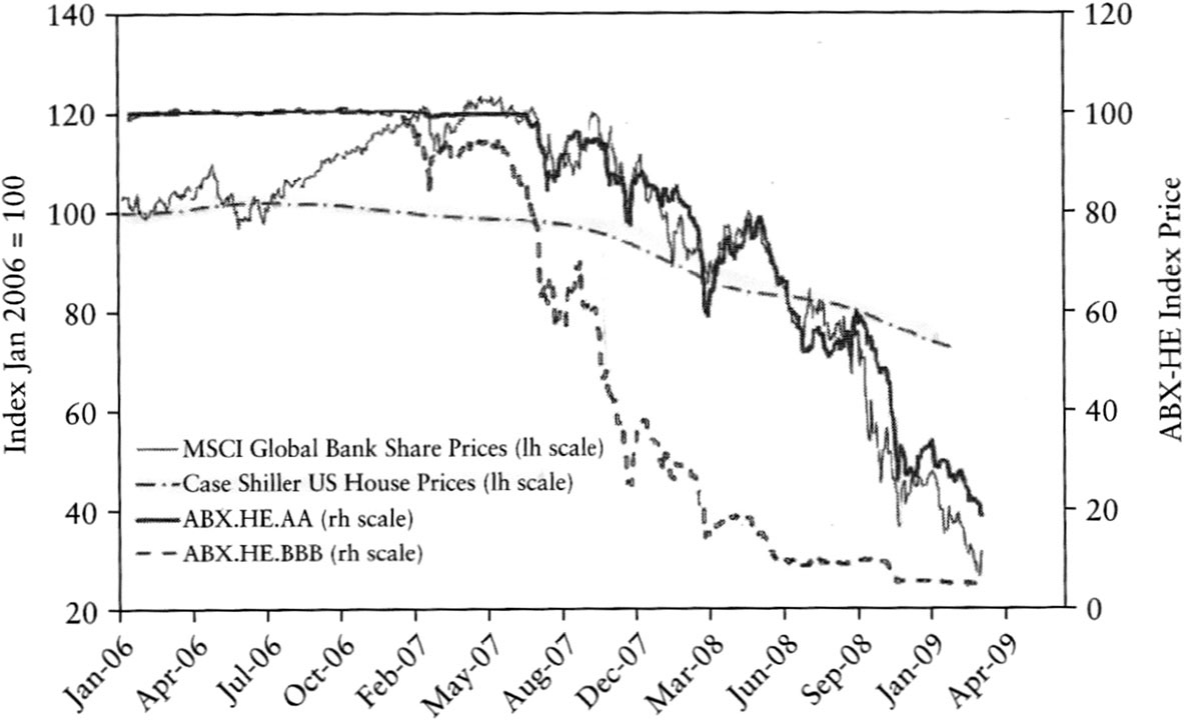
US House prices, ABX indices, and share prices of Global Banks. Source: Milne (2009, p. 201)

The logic is that ‘social contagion’ helped fuel a bubble in house prices; and this underpinned the boom in subprime lending, where borrowers were encouraged to share in the expected capital gains and the CRAs helped to conceal the risk that was involved. As Holmstrom 2009, p. 267) pointed out, however, the ‘dynamic credit enhancement’ that underpinned subprime lending could only work if house prices -- already buoyed by a bubble -- continued to rise incessantly: so the subprime mortgage boom was ultimately not sustainable. When house prices stopped rising, this, indeed, had a devastating impact on the value of securities backed by mortgages issued during the bubble. Figure 6 shows the prompt collapse in the price indices for AA and BBB packages of such securities (by 80% and over 90% respectively, rh scale) after house prices had peaked – and how this impacted a broad index of bank shares.

Both in his address on “Narrative Economics” to the American Economic Association and in his monograph with the same title, (Shiller 2017; Shiller 2019) Robert Shiller refers to the SIR model of Kermack and McKendrick (1927) as one that might be applied to social contagion. As it happens, however, a variant of the SIR model has already been applied to the housing market using the Case-Shiller indices of house prices, namely Burnside et al. (2016), hereafter BER.^23^ For the convenience of the reader, a brief outline of the SIR epidemic model and a summary of the BER approach – along with a ‘health warning’ - are provided in the Annex.

Thus, while not explicitly acknowledged by Shiller himself, an account resembling that being used for the Covid crisis^24^ has been used to explain the ‘epidemic of social contagion’ in the housing market. This social epidemic - together with the disingenuous role of the CRAs – could have played a key role in explaining how the financial system got to what has been called the ‘fooling equilibrium’ of the FCIC narrative.

### Significant Externalities in Play

As each infected person can pass the virus on to others – to three others in the early days of the UK first wave, for example - Covid involves significant health externalities. Even when private action to avoid infection is allowed for,^25^ as it fails fully to take account of the health of others, there is need for public policy to limit the externality (with interventions to mandate face-coverings, social distancing, lockdowns, etc.) until a vaccine is found. That national lockdowns have been imposed – and re-imposed – in so many countries is testimony of how far policy-makers are willing to go to check the externality of contagious spread.

If it encourages risk-shifting by banks with limited liability, the drive to maximise the ROE will also generate significant externalities - in the form of unanticipated losses to creditors and disruption to borrowers.^26^ Subsequent to the crisis of 2008–9, significant fines have been imposed on banks (for misleading others as to the quality of the MBS they sold) and on CRAs (for deliberate over-rating).^27^ Such fines undoubtedly hit the ROE, but getting corporations to internalise social costs appears to require targeting key decision-makers, rather than shareholders in general.^28^

As Paul Romer (Romer 2012) argued in the wake of the financial crisis, however:

There are workable alternatives to the legalistic, process-oriented approach that characterizes the current financial regulatory system in the United States. These alternatives^29^ give individuals responsibility for making decisions and hold them accountable.

In this spirit, Goodhart and Lastra (2020) propose that key decision-makers in banks and significant financial institutions be made personally liable for downside losses ‘so as to shift the costs of failure back to those who have responsibility for taking corporate decisions’. Zingales (2020) argues likewise that a fiduciary duty towards society be imposed (on all large corporations, not just big banks) with the board personally responsible for damaging externalities.

Such proposals appear more consistent with the sceptical assessment of the conduct of limited liability corporations made by the FCIC, than with the endorsement of their financial innovations offered by G&K, as cited above.

### The Role of Vaccine: Is there an Analogue Available for ‘Social Contagion’?

Vaccines - now being approved for general release against Covid-19 - promise an accelerated transition to ‘herd immunity’, as shown graphically for the SIR model in the Annex. This prompts the question: is there an analogue for epidemics of social contagion?

For BER, where the bubbles arise from uncertainty (as to whether renting is preferable to owning a house), the answer lies in the resolution of this uncertainty. How this might be achieved is not discussed, however. In a recent Bank of England paper, Haldane et al. (2020), epidemic models of social dynamics like BER are seen as cases where ill-informed ‘noise’ prevails; and the alternative proposed is one of ‘narrative entrepreneurship’ – of helping people make better sense of the economy and to form more reasonable expectations. Would it be stretching their argument too far to conclude that the Central Bank could supply the vaccine - reducing the ‘noise’ by appropriate sectoral intervention on credit under macro-prudential regulation, for example*?*

## Conclusion

In remarks on the Global Financial Crisis, Jon Danielsson makes a useful distinction between *exogenous* and *endogenous* risk. The former, as its name suggests, comes from outside, affecting agents within the system without being influenced by them. ‘Endogenous risk, on the other hand, is created by the interaction of economic agents, all with their own agendas, abilities, resources and biases’ (Danielsson 2019, p. 258).

In the narrative told by the scholars studied here, attention focuses on the vulnerability of the seemingly-efficient ‘supply chain’ of finance in the face of exogenous disturbances, be they unanticipated technology shocks (that damage bank portfolios) or ‘sunspots’ (that cause creditors to run). Endogenous risk seems effectively ruled out however, with assets held by banks perceived as riskless and self-regulation keeping managers honest.

At first blush, the Covid epidemic seems to support the idea of extraneous shocks being the driver of crisis. Yet, in the FCIC telling, the banking crisis is like what happens in Shakespeare’s *Tempest,* where it emerges that the fierce storm that opens the play was called up by Prospero, one of the players! For, in the Congressional narrative, it is the behaviour of the players themselves that generates much of the action. There are CRAs ready to mis-rate risky assets for a fee; shadow bankers seeing this as a way of shifting risk onto creditors despite the VaR rules in place; and creditors lending freely - until they find out what’s happening and respond with swingeing haircuts to bank collateral! Minsky famously argued that capitalist economies are unstable because of their financial markets and institutions. This narrative supports no such universal proposition: but it does sound a clear note of warning - that capitalist economies can be destabilised when financial markets and institutions put profits before purpose.^30^

Here, as in Rubinstein’s fable, the choice of narrative is left to the reader. But we can conclude on a positive note. For it appears that regulatory reaction to the near-collapse of shadow banking - by making the system more resilient to risk whatever its source – has put banks in better shape to handle the subsequent Covid pandemic!

## Figures and Tables

**Fig. 7:**
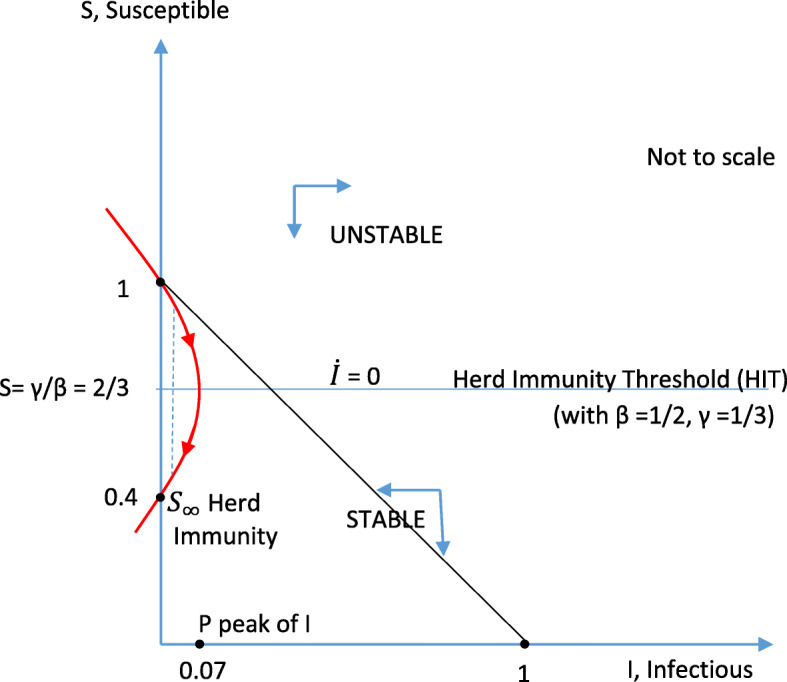
Epidemic unchecked - except by vaccine

## Annex: Medical and Social Epidemics

### The SIR Model for Spread of Disease (Smith and Moore 2001)

There are three groups in the population, Susceptible, S, Infectious, I, and Recovered, R, which evolve as follows:

1 $$ \dot{S}=-\upbeta S\ I $$

2 $$ \dot{I}=\upbeta S\ I-\upgamma I $$

3 $$ \dot{R}=\upgamma I $$

where β is the number of close contacts per period between susceptibles and those already infected; γ measures the rate at which the infected population ceases to be infectious; so β/γ  is the ‘reproduction ratio’ - the initial rate of spread; and the constant population is normalised at 1.

The dynamics of S and I are shown in the phase diagram below, along with the peak level of Infected population (P),the Herd Immunity Threshold (HIT) and the specific level of Herd immunity (*S*_∞_) where infections cease in the long run with no intervention. The parameters used are based on the Hong Kong flu epidemic in New York City in the late 1960’s.

No indications are given here for non-pharmaceutical interventions such as social distancing, lockdowns, and test/trace /isolate measures^31^; but the dashed line illustrates the impact of a vaccine for about half the population that takes the susceptible population promptly below the Herd Immunity Threshold, without affecting the level, *S*_∞_, where infections finally cease. (More or less vaccination would affect this level) (Fig. 7).

### The Social Dynamics of BER, i.e. Burnside et al. (2016)

BER use a version of SIR, but it is considerably modified as follows:

All agents have identical tastes, but hold different beliefs about a possible change in the utility of home owning versus renting. The three groups are Vulnerable, Skeptical and Optimist, where the latter believe with confidence that the utility is going to increase by a factor x; while the other two believe there will be no change, of this Skeptics being more sure than the Vulnerable. When members of any group meet those of another, beliefs can be affected: in particular, those with greater confidence can persuade others to adopt their beliefs. Unlike the SIR model, where the transition from S is to I and then to R, in BER there is greater mingling; thus one can transfer from Vulnerable to either of the other two groups; and there is also switching between the groups.

Depending on parametric values of these switching probabilities, starting with very few of either Optimist or Skeptical, one can expect a *temporary boom* in house prices followed by a reversal as Skeptical beliefs prevail; or a *boom that is not reversed* where Optimistic beliefs prevail. This is when the uncertainty is unresolved. If it is resolved in favour of the increase *x*, house prices will go up to boom levels; if not then prices will revert to original values.

Easley and Kleinberg (Easley and Kleinberg 2010, chapter 21) have issued a health warning about using biological models to describe social contagion, on the grounds that the latter involve conscious decision-making while the former are based on random transmission. Is the notion of ‘strength of belief’ used by BER to govern the transmission of ideas an adequate response?

## Footnotes

1 ‘The word model sounds more scientific than the word fable or tale, but I think we are talking about the same thing.’ Rubinstein (2012, Chapter 1, p. 16).

2 These are investment or wholesale banks that operated “without the security of insured deposits that never run, without effective regulatory constraints on their leverage and without the ability to turn to the Fed if their financing evaporated.” Bernanke et al. (2019, p. 23).

3 Chapter 2 of Akerlof and Shiller (2015).

4 As in Shin (2010, Chapter 3, Fig. 3.2).

5 which, for given equity *e*, allows for greater holdings the less risk exposure per unit.

6 As formally the parameter *η* = 3*τ*/*z*^2^, where τ is a measure of risk tolerance taken as fixed, Shin (2010, p. 31).

7 so $$ ROE=\left(q-p\right)\frac{y}{e}=\frac{q-p}{p}\ \frac{py}{e}= Mean\ return\  on\  assets\ x\  leverage $$

8 ‘In 2007 Moody’s, Standard and Poor’s and Fitch [the leading US credit rating agencies] employed about 14,000 people between them, covering 95% of the rating market. Although the rating agencies call their ratings mere ‘opinions’, these opinions have legal power as far as pension funds, boards of trustees of charities and other institutions are required by law to hold only investment grade securities’. Sinn (2010, pp. 127,8).

9 Moody’s Corporation went public in 2000; and, ‘after the public listing, the company culture changed – it went “from [a culture] resembling a university academic department to one which values revenues at all costs”.’FCIC (2011, p. 207).

10 In the terminology of Akerlof and Shiller (2015).

11 If not, creditors can raise the rate they charge for funding, as in Adrian and Shin (2014).

12 It is for a two-point process, however, rather than the uniform density of Fig. 1.

13 As broadly illustrated in calibrations reported in Miller and Zhang (2019),  CEPR DP. 13834.

14 where the ‘haircut’ is the difference between the current market price of the collateral and the price at which it is sold to the creditor – for future repurchase.

15 With leverage for the big five US investment banks of about 30 before the crisis, Miller, Rastapana and Zhang (2018, Table 2, col. 5).

16 Note that, with precipitous panic, equilibrium may shift promptly from B to D, bypassing C.

17 In the simulations, 4% is the steady state value of interest paid to bank creditors, which matches the household rate of time preference (in order, presumably, that money be held in equilibrium).

18 The equity constraint takes the form QKb ≤ φN, where Q is the asset price, Kb is holding by banks, whose net worth is N.

19 In the absence of figures for the impact effect of the productivity shock *before assets change hands*, a point B has been inserted showing an initial price fall akin to the size of the shock.

20 Where the probability that an investment payoff will move more than three standard deviations from the mean is greater than that of a normal distribution.

21 In a setting of asymmetric information, such downside risk in investment portfolios can- by using derivatives - be concealed from outside observers for considerable periods of time, Foster and Young (2010), Sinn (2010).

22 “The names of the derivatives being used included IBG (I’ll Be Gone if it doesn’t work), and in Chicago, the O’Hare Option (buy a ticket departing from O’Hare International Airport: if the strategy fails, use it; if the strategy succeeds, tear up the ticket and return to the office). That such strategies were common enough in the industry as to have names suggests that not all traders were oblivious of the risks they were taking.” Rajan (2010, p. 139).

23 For this key reference, not to be found in Shiller’s own literature surveys, I am indebted to Marija Vukotic.

24 For the SIR model has, of course, been widely applied in studying the course of Covid-19 - with Ferguson et al. (2020) and Eichenbaum et al. (2020) being prominent examples from the UK and USA.

25 As in Eichenbaum et al. (2020), for example, where individuals work and shop less.

26 Aikman et al. (2015) describe three ‘games bankers play’ that can generate these externalities.

27 Details in Miller, Rastapana and Zhang (2018, pp. 103,104).

28 Why the legal settlements have, nevertheless, taken the form of ‘deferred prosecution agreements’ with the companies involved, rather than the criminal prosecution of high-level individuals, is discussed in Rakoff (2014) - with the contrast between the US and Iceland in this respect high-lighted in Miller (2019).

29 The approaches to safety at the Federal Aviation Authority, to macroeconomic stabilization at the Fed and to race relations in the army, are three examples cited.

30 The Icelandic banking collapse of 2008 provides a fascinating supporting act – described in detail by Johnsen 2014 using the findings of Iceland’s Special Investigation Commission to do so.

## References

[CR1] Adrian T, Shin HS (2014). Pro-cyclical leverage and value-at-risk. Rev Financ Stud.

[CR2] Aikman D, Haldane AG, Nelson BD (2015). Curbing the credit cycle. Econ J.

[CR3] Akerlof GA, Shiller RJ (2015). Phishing for Phools: the economics of manipulation and deception.

[CR4] Bernanke BS (2018) Financial panic and credit disruptions in the 2007–09 crisis. Brookings Institution https://www.brookings.edu/blog/ben-bernanke/2018/09/13/financial-panic-and-credit-disruptions-in-the-2007-09-crisis/. Accessed 3 Mar 2019

[CR5] Bernanke BS, Geithner TF, Paulson HM (2019). Firefighting: the financial crisis and its lessons.

[CR6] Bolton P, Dewatripont M (2005). Contract theory.

[CR7] Burnside C, Eichenbaum MS, Rebelo S (2016). Understanding booms and busts in housing markets. J Polit Econ.

[CR8] Cole HL, Kehoe TJ (2000). Self-Fulfilling Debt Crises. Rev Econ Stud.

[CR9] Danielsson J, Aliber RZ, Zoega G (2019). Financial policy after the crisis. The 2008 global financial crisis in retrospect.

[CR10] Easley D, Kleinberg J (2010). Networks, crowds, and markets: reasoning about a highly connected world.

[CR11] Eichenbaum MS, Rebelo S, Trabant M (2020) “The Macroeconomics of Epidemics”. NBER Working Paper Series, No. 26882, March

[CR12] Financial Crisis Inquiry Commission (FCIC) (2011). Final Report.

[CR13] Foster DP, Young P (2010). Gaming performance fees by portfolio managers. Q J Econ.

[CR14] Ferguson NM et al. (2020) Impact of Non-pharmaceutical Interventions (NPIs) to Reduce COVID-19 Mortality and Healthcare Demand, Imperial College, London, March. 10.25561/77482

[CR15] Gertler M, Kiyotaki N (2015). Banking, liquidity, and Bank runs in an infinite horizon economy. Am Econ Rev.

[CR16] Gertler M, Kiyotaki N, Prestipino A (2020). A Macroeconomic Model with Financial Panics. Rev Econ Stud.

[CR17] Gertler M, Kiyotaki N, Prestipino A, Taylor JB, Uhlig H (2016). Wholesale banking and Bank runs in macroeconomic modeling. Handbook of Macroeconomics. Vol. 2B.

[CR18] Goodhart CAE, Lastra RM (2020). Equity Finance: Matching Liability to Power. J Financ Regul.

[CR19] Haldane A, Macaulay A, McMahon M (2020) The 3 E’s of central bank communication with the public. Bank of England. Staff Working Paper no. 847, January

[CR20] Holmstrom B (2009). Commentary: “the panic of 2007”. Maintaining Stability in a Changing Financial System.

[CR21] Johnsen G (2014). Bringing down the banking system: lessons from Iceland.

[CR22] Kermack WO, McKendrick AG (1927). A contribution to the mathematical theory of epidemics. Proc R Soc.

[CR23] Miller M, Zhang L (2019) Externalities and financial crisis – enough to cause collapse? CEPR DP 13834

[CR24] Miller M, Aliber RZ, Zoega G (2019). The financial alchemy that failed. The 2008 global financial crisis in retrospect.

[CR25] Miller M, Rastapana S, Zhang L (2018) The blind monks and the elephant: contrasting narratives of financial crisis. Manch Sch 86(S1):83–109 September. 10.1111/manc.12236

[CR26] Milne A (2009). The fall of the house of credit: what went wrong in banking and what can be done to repair the damage?.

[CR27] Moll B (2020) Lockdowns in SIR models. Technical report, LSE (May) https://benjaminmoll.com/wp-content/uploads/2020/05/SIR_notes.pdf. Accessed 21 May 2020

[CR28] Rajan RG (2010). Fault Lines.

[CR29] Rajan RG (2005). Has financial development made the world riskier? The Greenspan era: lessons for the future.

[CR30] Rakoff JS (2014) The financial crisis: why have no high-level executives been prosecuted? The New York Review of Books (January 9) Reference confirmed

[CR31] Romer P, Blanchard (2012). Process, responsibility and Myron’s law. In the Wake of the Crisis: Leading Economists Reassess Policy.

[CR32] Rubinstein A (2012). Economic Fables.

[CR33] Shiller RJ (2019). Narrative Economics.

[CR34] Shiller RJ (2017). Narrative economics. Am Econ Rev.

[CR35] Shiller RJ (2008). The subprime solution.

[CR36] Shin HS (2010). Risk and liquidity.

[CR37] Sinn HW (2010). Casino capitalism: how the financial crisis came about and what needs to be done now.

[CR38] Smith D, Moore L (2001) The SIR model for spread of disease. J Online Math Applic. https://www.maa.org/press/periodicals/loci/joma/the-sir-model-forspread-of-disease. Accessed 21 May 2020

[CR39] Zingales L (2020) Which capitalism? Markus’ academy webinar, Princeton Bendheim center (Oct 8)

